# Sustainability and Long Term-Tenure: Lion Trophy Hunting in Tanzania

**DOI:** 10.1371/journal.pone.0162610

**Published:** 2016-09-20

**Authors:** Henry Brink, Robert J. Smith, Kirsten Skinner, Nigel Leader-Williams

**Affiliations:** 1 Selous Lion Project, PO Box 34514, Dar es Salaam, Tanzania; 2 Durrell Institute of Conservation and Ecology, University of Kent, Canterbury, Kent, CT2 7NR, United Kingdom; 3 School of Geography, Planning and Environmental Management, University of Queensland, Brisbane, QLD 4072, Australia; 4 Department of Geography, University of Cambridge, Downing Place, Cambridge, CB2 3EN, United Kingdom; University of Illinois at Urbana-Champaign, UNITED STATES

## Abstract

It is argued that trophy hunting of large, charismatic mammal species can have considerable conservation benefits but only if undertaken sustainably. Social-ecological theory suggests such sustainability only results from developing governance systems that balance financial and biological requirements. Here we use lion (*Panthera leo*) trophy hunting data from Tanzania to investigate how resource ownership patterns influence hunting revenue and offtake levels. Tanzania contains up to half of the global population of free-ranging lions and is also the main location for lion trophy hunting in Africa. However, there are concerns that current hunting levels are unsustainable. The lion hunting industry in Tanzania is run by the private sector, although the government leases each hunting block to companies, enforces hunting regulation, and allocates them a species-specific annual quota per block. The length of these leases varies and theories surrounding property rights and tenure suggest hunting levels would be less sustainable in blocks experiencing a high turnover of short-term leases. We explored this issue using lion data collected from 1996 to 2008 in the Selous Game Reserve (SGR), the most important trophy hunting destination in Tanzania. We found that blocks in SGR with the highest lion hunting offtake were also those that experienced the steepest declines in trophy offtake. In addition, we found this high hunting offtake and the resultant offtake decline tended to be in blocks under short-term tenure. In contrast, lion hunting levels in blocks under long-term tenure matched more closely the recommended sustainable offtake of 0.92 lions per 1000 km^2^. However, annual financial returns were higher from blocks under short-term tenure, providing $133 per km^2^ of government revenue as compared to $62 per km^2^ from long-term tenure blocks. Our results provide evidence for the importance of property rights in conservation, and support calls for an overhaul of the system in Tanzania by developing competitive market-based approaches for block allocation based on long-term tenure of ten years.

## Introduction

Biodiversity conservation outcomes are closely related to the rules and institutions that govern the use of natural resources [1–3]. Since the 20^th^ century, statutory protected areas have been a cornerstone of biodiversity conservation strategies for most countries [4–6]. However, operating budgets for these protected areas in developing countries cover an average of 30% of their costs [7] and society seems reluctant to cover the shortfall of conserving biodiversity [8–11]. Thus, there is an increasing recognition of the role of the private sector in funding and managing conservation [12–14].

In southern and eastern Africa, a prime example of the private sector playing this role comes from the organised hunting of wild animals by tourists for sport or trophies [15–18]. Areas set aside for the hunting of big game animals protect habitats that might otherwise be converted to agriculture [19], protect populations of large mammals [20,21], and can benefit local people [22,23]. However, exploitation of a species always has the potential to reduce populations to levels where hunting is no longer profitable, or in extreme cases cause population extinctions [4,24–26], and legal hunting can have unintended knock on effects by encouraging illegal hunting [27]. This means that trophy hunting systems need to be developed with care, so they encourage the sustainable use of the target species [28]. One key aspect is providing secure resource tenure [29–31] and so conservationists are increasingly concerned with governance dynamics and the need for institutional reform [32–35].

These issues of ownership and tenure are especially relevant for Tanzania, a country that is hugely important for wildlife conservation and a prime destination for trophy hunters [18,36]. The Tanzanian government has long recognised the global importance of its biodiversity and has given protected area status to 37% of the country’s land surface [25]. Trophy hunting is permitted in all protected areas in Tanzania, apart from the National Parks and the Ngorongoro Conservation Area. Therefore, hunting occurs on some 305,000 km^2^, or some 86% of protected land. Tanzania is recognised for its high quality trophy hunting opportunities [37] and this remains a principal source of income for vast areas of the country [38]. Moreover, the hunting industry has demonstrated an impressive growth in recent years and is an important source of foreign exchange [18,39].

**Fig 1:**
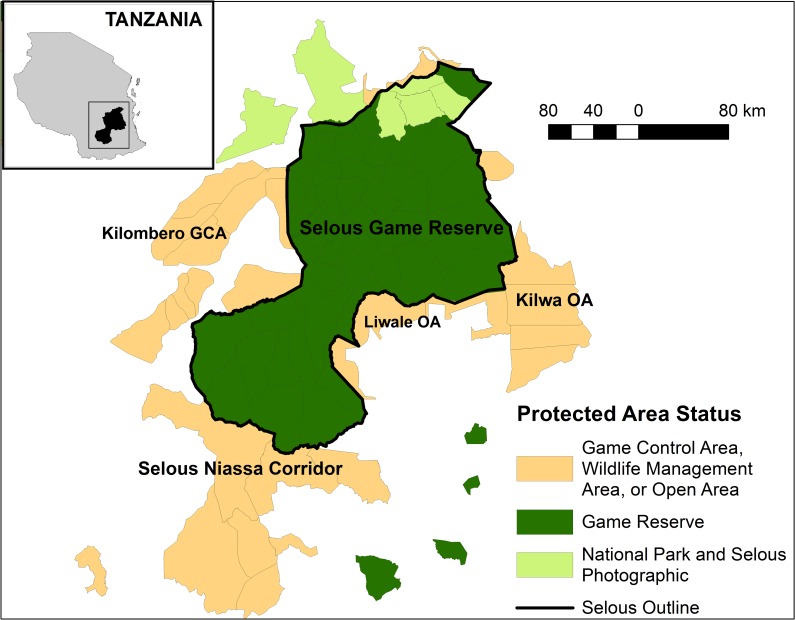
Selous Game Reserve and bordering hunting blocks and national parks.

Nonetheless, a review of protected area tenure arrangements in Tanzania noted that all land is ultimately owned by the state, and may only be leased by companies/individuals for set periods of time, no longer than 99 years [40]. Hunting companies lease one or several hunting blocks, which are segments of land designated as a Game Reserve, Game Control Area, Wildlife Management Area, or Open Area (see Fig 1), and the company is allocated a species-specific quota for each block for the hunting season [41]. A portion of this quota is then offered to clients by the hunting company, who stay at hunting camps for 1, 2 or 3 week periods depending on species the client wishes to hunt. Clients wishing to hunt lion are required to purchase a 21-day safari. Clients may fly between different hunting blocks leased by the same company in order to hunt different species only found in certain areas. This means that the Tanzanian government has a lot of control over where and for how long different trophy hunting companies are allowed to operate, as well as the number of animals that can be hunted by tourists. Thus, their policies and actions could have a large influence on the ecological and financial sustainability of the industry.

Despite the influence of a national policy on the sustainability of trophy hunting in Tanzania and its major conservation role, very little information is available on the industry and many aspects are shrouded in secrecy [16,18]. In particular, many of the concessions are leased to local companies that do not have the capacity to market their hunting opportunities, which leads to a system of subleasing, mostly to foreign non-resident professional hunters. This has implications for revenue collection because these hunting opportunities are often cheaply subleased and much of the generated income never enters Tanzania, and so cannot be taxed by the Tanzania Revenue Authority [38]. Furthermore, the blocks are sub-leased for short periods, which may encourage their over-utilization. Such over-utilization is a particular concern for lion conservation in Tanzania, as the country supports between a quarter and half of the remaining free-ranging lions in the world [25,42]. In addition, Tanzania is the most important destination for sport hunting of lions, exporting an average of 243 wild lion trophies per year between 1996 and 2006, compared to 96/yr from Zimbabwe, and 55/yr from Zambia, while no other country exported more than 20/yr [43].

One of the most important sites for lion trophy hunting is the Selous Game Reserve (SGR), a World Heritage Site in the south east of Tanzania. SGR has developed a considerable reputation as a tourist hunting destination [44] and contains an internationally important lion population [45]. Lion populations are declining in most of their range across Africa [42,46,47] and trophy hunting has been shown to be in need of reform [18,25]. Therefore, here we use data from SGR to investigate the factors that determine lion trophy hunting sustainability. We investigate the extent to which lion hunting offtake influences offtake over time and resultant government revenue. We also look at the differences in lion hunting offtake and government revenue between hunting blocks with short-term and long-term leases.

## Material and Methods

We are grateful for permission and constructive advice from the Tanzania Wildlife Research Institute (TAWIRI), Commission for Science and Technology (COSTECH) and the Wildlife Division (WD) of the Ministry of Natural Resources and Tourism to carry out research in Selous Game Reserve.

### Study Area

**Fig 2:**
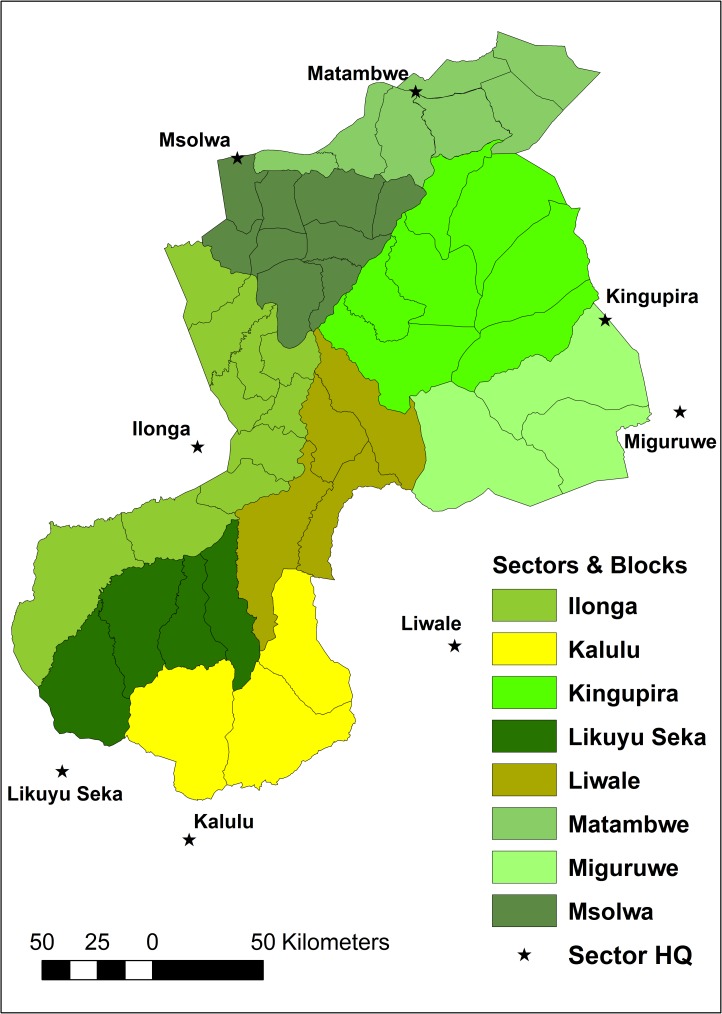
Map of sectors and blocks of Selous Game Reserve.

The Selous ecosystem is an area of 96,643 km^2^ and contains 64 hunting blocks. Of these, 43 blocks are within SGR, seven are in areas surrounding SGR and have been hunted since the 1990s, while another 14 have more recently been designated and have only been hunted since 2002 [41]. The Selous ecosystem also contains conservation land where trophy hunting does not take place (National Parks), including four blocks within SGR that are set aside for photographic tourism (Fig 1). For management purposes SGR is divided into eight sectors (Fig 2), which we use when reporting some of the results.

SGR is surrounded by a network of different protected areas, namely: National Parks, Game Control Areas, and Open Areas and Wildlife Management Areas (Fig 1). The distinction between hunting blocks on Game Control Areas, Wildlife Management Areas and Open Areas is not clear cut, as all allow for human settlement and wildlife to coexist, and hunting is only permitted under licence. Instead, they reflect when the blocks were established in relation to different phases of development in national wildlife policies. Game Control Areas are the oldest, and most were set-up prior to the early 1990s. The Wildlife Management Areas reflect Tanzania’s attempt to introduce community-based management of wildlife in the late 1990s, with the idea that control of and benefits from wildlife would be decentralised to communities living in the area. The re-designation of some Wildlife Management Areas as Open Areas and the designation of new hunting blocks in 2004/5 as Open Areas reflect both the central government’s move away from devolving power to local communities and the preference of hunting companies to deal with one central authority [34]. Fees paid by the hunting companies to the government or local communities are used to provide law enforcement and infrastructure development. Nonetheless most hunting companies also privately maintain infrastructure (e.g. roads) and carry out anti-poaching activities within their blocks.

### Block Boundary Data

We obtained the digital boundary polygons files of the SGR blocks from the Tanzania Wildlife Research Institute and the Selous Conservation Project, which was funded by the Organization for German Technical Cooperation (GTZ). The Selous Conservation Project data were from 2003, while the Tanzania Wildlife Research Institute data were more up-to-date and showed boundaries from 2009. We conducted field visits from June 2006 to August 2009 to the different sectors of SGR to investigate the accuracy of these layers and updated them as necessary. All spatial data were imported into ArcGIS version 9.3 (ESRI Inc., Redlands, CA) for analysis.

### Block Tenure and Hunting Fees

We obtained information on which companies leased which block, and on hunting fees, from the Wildlife Division of Tanzania’s Ministry of Natural Resources and Tourism. Information on who owned a specific company, and for how long, including information on subleasing, was acquired from informants within the hunting industry. We acquired data for the average government income per block and the source of these payments (e.g. block fees or trophy fees) from the Selous Conservation Project (listed in [38]). These data on income were only available for blocks within SGR and from 1996 to 2003. We defined long-term blocks as those that were leased by the same owner and company for at least 10 years, although most of these blocks have been in the same hands since the early 1990s (>15 years). In contrast short-term blocks have changed leasers at least once in the 10-year period, with most having changed hands several times in this period or having been subleased to several non-resident professional hunters.

### Lion Hunting Offtake Data

Data on number of lions killed in each hunting block of SGR were provided by the CITES office at the Wildlife Division Headquarters. In Tanzania, only male lions may be hunted. This hunting offtake data were much more complete within SGR, as compared to the rest of Tanzania, due to the activities of conservation development projects (Planning and Assessment for Wildlife Management & Selous Conservation Project [35,36,39]). We also collected background information on lion mortality trends through one-to-one discussions with sector wardens, company owners, and professional hunters. We did not use data on lion population trends because, while these data have been calculated for the photographic tourism area of SGR [45], they are not available for Tanzania’s hunting blocks. However, previous researchers have suggested that hunting offtake data are a proxy for this population data, principally because hunting companies put a large amount of effort into finding lion trophies, and so any changes in the underlying population are reflected in the number of lions hunted [25,43].

### Data Analysis

We carried out data exploration and analysis using SPSS (version 23.0, IBM SPSS Inc) and ArcGIS. To measure hunting offtake per block, we calculated the mean annual lion offtake per block per 1000 km^2^ from 1996–2008. We calculated for 64 blocks the change in the annual lion hunting offtake by using linear regression analysis to model changes over time using data on the number of lions hunted per year between 1996 and 2008. We then used linear regression to investigate whether annual rate of change in hunting offtake was influenced by annual lion hunting offtake for the 43 blocks within SGR, having log-transformed the hunting offtake metric to meet the assumptions of the test. At the sector management level, we used Kruskal-Wallis tests to see if there were any differences per sector in government income, hunting offtake, or proportional rate of change in hunting. We also used a Spearman’s rank test to measure the correlation between government income per km^2^ per block and annual rate of change in lion hunting offtake. We used Mann-Whitney U tests to investigate differences between 26 long-term blocks and 17 short-term blocks in terms of hunting offtake, annual change in lion offtake and government revenue. We did not include data from one hunting block, K5, for the analyses of lion hunting offtake, as the lessee banned the hunting of lions in the block from 2002 onwards. We did use data from K5 in the analysis of income per SGR sector, as the lessee continued to hunt other species during the study period.

## Results

### Block Tenure

Twenty hunting companies were listed as leasing blocks in SGR between 1995 and 2009. Twenty-six blocks were under long-term tenure and 17 blocks were under short-term tenure. Data on government income per block was only available for blocks within SGR and from 1996 to 2003 (listed in [38]). During this period government income from hunting activities was dependent on six different fees. The two key fees are the trophy fee, which is the amount paid when a targeted animal is killed, and the block fee, which is the fee paid annually by a company to lease a block. From 1996 to 2003, government income was heavily reliant on trophy fees (accounting for 59% of government income from hunting). The lion trophy fees accounted for almost ten percent of the overall wildlife trophy fees. Block leases in 2003 were only $7500 per block, regardless of size, and therefore only accounted for 11% of the government income from hunting. Block fees increased to $12,000 in 2006, and then $27,000 in 2008, and up to $60,000 in 2011.

### Lion Hunting Offtake and Hunting Trends

**Fig 3:**
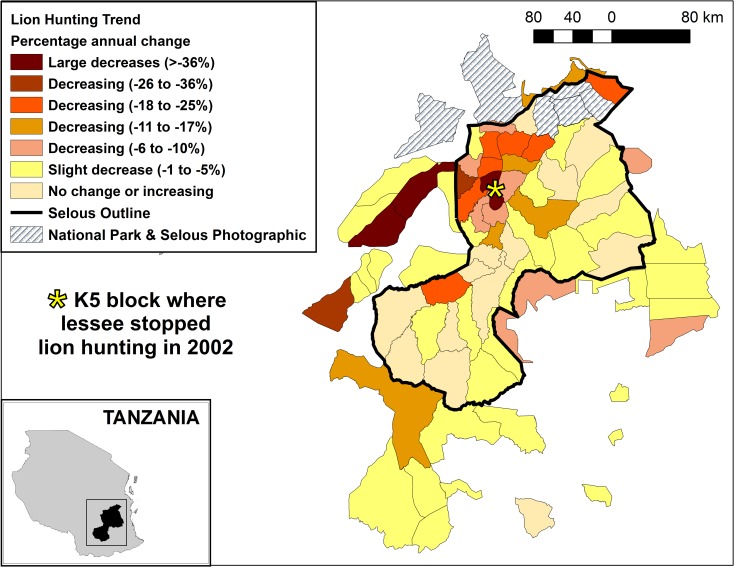
Map showing annual change in annual lion hunting (1996–2008) in Selous Game Reserve and surrounding blocks.

**Fig 4:**
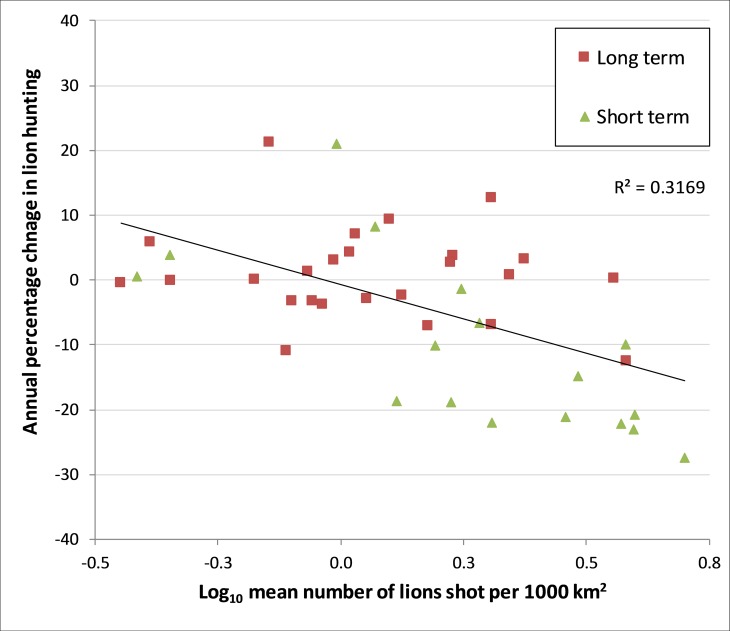
Long-term versus short-term tenure in Selous Game Reserve, shown as the annual rate of change in hunting offtake (%) and lion hunting offtake.

Lion hunting offtake (number of lions shot per 1000 km^2^ per annum) was higher in blocks inside than outside SGR (mean inside 1.8 ± 1.2; mean outside 1.1 ± 0.8). Offtake was especially high in the north western part of SGR, within the Msolwa, Ilonga and Matambwe sectors (Table 1). The annual change in lion hunting offtake was negatively related to the log_10_ number of lions shot per 1000 km^2^ per block (N = 42, R^2^ = 0.317, p < 0.001; Figs 3 and 4, and Table 1), with the model predicting that offtake is sustainable (i.e. where annual change is 0 or above) when hunting is ≤ 0.92 lions per 1000 km^2^.

**Table 1:** Data on lion hunting offtake per 1000km^2^, change in hunting offtake, and government income per km^2^ from within Selous Game Reserve by sector.

Sector	No. of Hunting Blocks	Total Area of Sector (km^2^)	Average Lion Hunting Offtake	Annual Change in Hunting Offtake (%)	Average Government Income (US$)
Ilonga	10	7521	2.25 ± 1.48	-6%	130.16 ± 82.50
Kalulu	3	4989	0.86 ± 0.19	0%	26.57 ± 1.36
Kingupira	7	9345	1.82 ± 0.97	0%	65.08 ± 20.42
Likuyu Seka	4	5025	1.36 ± 0.72	6%	44.85 ± 22.76
Liwale	4	4716	0.67 ± 0.28	3%	35.30 ± 17.32
Matambwe	3	1738	2.22 ± 1.53	-7%	134.09 ± 33.95
Miguruwe	3	6124	0.86 ± 0.43	1%	34.73 ± 13.56
Msolwa	9	4642	2.38 ± 1.14	-18%	135.27 ± 51.59
Total	43	44100	1.55 ± 0.70	-	75.75 ± 48.86

Mean lion hunting offtake (± standard deviation) from 1996 to 2008, annual rate of change (%) in hunting from 1996 to 2008, and mean government income per km^2^ from 1996 to 2003 (± standard deviation). See Tables A & B in S1 File for the above data on a block by block basis.

### Sectors within SGR and Government Income from Trophy Hunting

There was a difference in the income per SGR sector (N = 43, H = 27.40, 7 d.f., p < 0.001), proportional annual change in lion offtake per sector (N = 43, H = 14.80, 7 d.f., p = 0.039), and hunting offtake per sector (N = 43, H = 14.97, 7 d.f., p = 0.036). The sectors with the highest lion hunting offtake experienced the steepest declines in hunting offtake from 1996–2008, but provided the government with the greatest income per km^2^ from 1996 to 2003 (Table 1). Government income per km^2^ per block of SGR was negatively correlated with the annual rate of change in lion hunting offtake (N = 43, *r*_*s*_ = -0.62, p < 0.001). That is, blocks with the greatest reduction in lion offtake from 1996–2008 generated the highest amount of government income per km^2^ per annum from 1996–2003.

### Block Tenure: Long-Term Versus Short-Term

**Fig 5:**
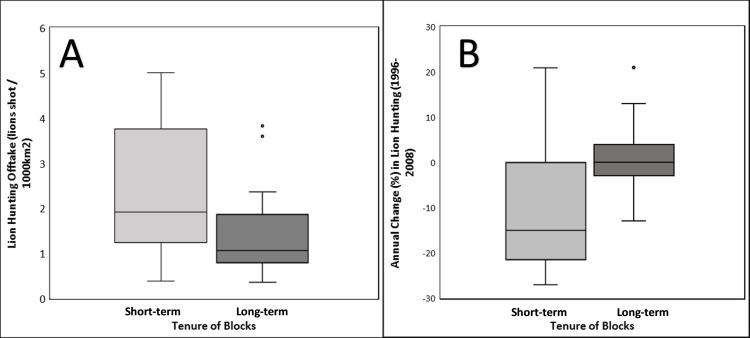
Fig 5A. Lion hunting offtake (lions shot per 1000km^2^), long-term versus short-term block tenure; Fig 5B. Annual rate of change in lion hunting offtake from 1996 to 2008, long-term versus short-term tenure.

Long-term blocks had a lower hunting offtake (long-term mean 1.41 ± 0.09 and short-term mean 2.33 ± 1.38; N = 42, Z = -2.371, p = 0.018; Figs 4 and 5A) and annual change in lion offtake (long-term mean -0.80 ± 10.99% and short-term mean -10.76 ± 13.36%; N = 42, Z = -2.989, p = 0 .003; Figs 4 and 5B). Long-term blocks also provided the government with less revenue (long-term mean per km^2^ was $62.20 ± 41.66; short-term mean per km^2^ was $133.17 ± 71.20; N = 43, Z = -3.577, p < 0.001).

## Discussion

Length of tenure has important implications for the sustainability of natural resource utilization. Managers of areas under long-term tenure are more likely to maintain the long view and husband their resources [29,48,49]. By contrast, the highest offtakes of hunted lion, and the highest levels of income for the government, were recorded in blocks under short-term tenure. However, this short-termism is driving the over-hunting of lions, leading to declines in the lion population in these hunting blocks. The following sections will discuss this issue in more detail, ending with recommendations on reforming the system.

### Lion Conservation and Trophy Hunting Trends

The primary threats to large felids across Africa are thought to be retaliatory killing and habitat loss [42,50–52]. However, over-hunting is also a possible cause of concern, especially in felid species like the lion where infanticide is common [41,53]. Within the Selous ecosystem but outside SGR, it is clear that decreases in lion populations have occurred as a result of conflict with people [47,54]. However, only recently has enough evidence been gathered to suggest that trophy hunting of lions is having a negative impact on populations [25,43,55,56]. Lion trophy hunting specifically targets adult males and sport hunters are extremely efficient in locating their quarry. This has large impacts because the males that replace the hunted individuals in the pride kill any cubs they have not fathered [25]. Recent research from Zambia suggests that lion trophy hunting could be sustainable with a strategy that combines periods of recovery or no hunting, a minimum age of at least seven years for trophy lions, and a quota of 0.5 lions per 1000km^2^ [57]. Similarly, research from West Africa suggests a quota of 1 lion per 1000km^2^ would be sustainable [58].

In terms of lion trophy hunting in Tanzania, and in SGR in particular, hunting levels peaked in the late 1990s and declined by up to 50% in the following decade [25,43]. The SGR blocks with the highest lion harvests per 1000 km^2^ showed the steepest declines in hunting (Figs 3 and 4). Trophy hunting since the late 1990s has had a negative impact on lion populations in SGR, and research from across Tanzania suggests that the sustainable lion trophy hunting quota is one lion per 1000km^2^ for SGR and half that for the rest of the country [25]. However, to achieve this balance in the future it is important to understand why lion trophy hunting in some areas of SGR is unsustainable and what is driving this process.

### Government Income per Block

In Tanzania, the government has relied on trophy fees to raise income from trophy hunting, rather than raising revenue through block fees [59]. Government regulations also stipulate that companies have to achieve 40% of their overall quota or face penalties [16,38]. Hunting companies have been happy with this system, as it is easier to pass trophy fee costs to clients than it is to transfer costs like block fees. However, the trophy fees for lion are higher than for other animals ($4900/lion in 2009) and this creates pressure for setting higher quotas, as increasing the number of lion on quota greatly increases government income. This leads to higher lion hunting offtakes and then declines in offtake. Thus, the blocks with the greatest declines in lion trophy hunting from 1996–2008 were the same blocks that provided the government with the most income per km^2^ from 1996–2003.

Length of tenure is closely associated with levels of income generated for the government. Hunting blocks under short-term tenure (and in most cases subleased blocks) provided almost twice the revenue per km^2^ than the blocks that have been under long-term stewardship ($133 per km^2^ short-term to $62 per km^2^ long-term). The Msolwa, Ilonga and Matambwe sectors of SGR are those where the most subleasing occurs, and they provided the most revenue per km^2^ to government from 1996–2003 (see Table 1). However, these are also the sectors with the highest lion hunting offtake and the greatest declines in annual lion hunting offtake from 1996–2008. Furthermore, over-hunting can have impacts on neighbouring unhunted areas [55], where the empty territories, caused through hunting, act as population sinks, drawing in lions from neighbouring areas. This suggests the high revenue in the short-term blocks depended in part on there being long-term blocks with better managed lion populations.

### Block Tenure

Hunting companies that have retained the same hunting blocks over 20 years are thought to take a long-term view of managing their hunting opportunities [60]. This relationship is clearly highlighted by our lion trophy hunting data. That is, when compared to short-term blocks, long-term hunting blocks experience a lower offtake of hunted lions and a smaller annual change in lion offtake between 1996–2008 (Figs 4 & 5). Ideally, our analysis would have included data on lion densities and hunting effort per hunting block, as this would have allowed a more in-depth assessment of lion hunting sustainability. However, such data were not available and so instead we used results from a previous Tanzania-wide study on lion hunting levels and population changes to assess the sustainability of hunting in SGR [25]. We found that the lion hunting offtake in the long-term blocks in SGR matches the sustainable level of one lion per 1000 km^2^ recommended in the previous study. Moreover, our results support this recommendation, as offtake has been relatively constant from 1996–2008. In contrast, hunting offtake in the short-term blocks is almost double the recommended level and offtake has declined (Fig 5).

Data from specific blocks under short-term ownership also provide evidence of the relationship between tenure and offtake. These blocks were predominantly on the western side of SGR in the Msolwa and Ilonga sectors. They experienced very high hunting offtakes in the late 1990s and then subsequent declines in hunting offtake in the early 2000s. However, by 2009 parts of the Msolwa sector supported some of the highest lion densities in SGR [45]. It is thought that the over-harvesting of the late 1990s had led to a scarcity of lions to hunt, which made it difficult to attract clients to the blocks and allowed lion populations to recover by 2009. The decision by the lessee in 2002 to stop hunting lions in a block in the heart of the Msolwa sector (K5 on Fig 3) probably helped this recovery. Over the last few years several blocks that were over-utilised have changed hands, to what is hoped will be more responsible owners.

## Conclusions and Recommendations

Trophy hunting has had a negative impact on lion populations in SGR since the late 1990s, and research suggests the sustainable lion trophy hunting quota is one lion per 1000km^2^ in SGR [25]. Applying this in SGR would have only led to a slight decrease in the 2008 overall lion hunting offtake, from ~50 lions a year to ~45 lions a year, but would have resulted in a much more even spread in lion hunting across SGR between sectors and blocks under different lengths of tenure.

The blocks in SGR with the highest lion hunting offtakes also experienced the steepest declines in trophy offtake from 1996 to 2008 and tended to be under short-term tenure. These high pressure hunting blocks, however, brought in the greatest amount of revenue for the government per km^2^ of area. The solution to this problem is not new, but involves adopting the 1995 Policy and Management Plan for Tourist Hunting [61], which was accepted by the then Director of Wildlife, but has yet to be implemented [60]. This would allocate hunting blocks through market-based competitive bidding (i.e. an auction) with a long-term lease of ten years, thereby reducing the importance of trophy fees and moving away from the current pay-as-you-use approach. Such recommendations remain pertinent, as Tanzania offered 28 new hunting blocks in May 2015, each on a three year lease [62], with a plan to assess all hunting blocks and potentially re-allocate them in 2018. The main drivers for such re-allocations seem to be to generate increased revenue for the government and to increase the number of Tanzanian nationals leasing blocks. Such goals are laudable but the allocation process must also involve increased transparency in block allocation, income generation, hunting offtake and quota setting. Otherwise, unsustainable hunting offtakes will continue.

## Supporting Information

S1 File Table A: Data on mean lion hunting offtake (± standard deviation), annual rate of change in hunting from 1996 to 2008, and government income per km^2^ from 1996 to 2003 from within Selous Game Reserve by hunting block. Table B: Data on lion hunting offtake (± standard deviation) and annual rate of change in blocks outside Selous Game Reserve (1996–2008). Table C: Maximum offtake (1996–99), average offtake and quota per block (1996–2008) in SGR. Figure A: Average number of lions shot per block in Selous Game Reserve and the average lion hunting quota per block per year.
(PDF)
