# Supplementary material for: Sustainability and Long Term-Tenure: Lion Trophy Hunting in Tanzania
Source: PLoS One. 2016 Sep 20;11(9):e0162610. doi: 10.1371/journal.pone.0162610 (PMC5029936; doi:10.1371/journal.pone.0162610)
Supplement: S1 File — Table A: Data on mean lion hunting offtake (± standard deviation), annual rate of change in hunting from 1996 to 2008, and government income per km2 from 1996 to 2003 from within Selous Game Reserve by hunting block. Table B: Data on lion hunting offtake (± standard deviation) and annual rate of change in blocks outside Selous Game Reserve (1996–2008). Table C: Maximum offtake (1996–99), average offtake and quota per block (1996–2008) in SGR. Figure A: Average number of lions shot per block in Selous Game Reserve and the average lion hunting quota per block per year. (PDF) [file pone.0162610.s001.pdf]

Supporting Information:

Table A: Data on mean lion hunting offtake (± standard deviation), annual rate of change in hunting from 1996 to 2008, and government income per km^2 from 1996 to 2003 from within Selous Game Reserve by hunting block.

Hunting Block Name	Sector	Area (km^2)	Mean lion shot per block	± *SD*	Lion Offtake (lion per 1000km^2)	% Change in Offtake	Govt Income ($ per km^2)
Selous IH1	Ilonga	425	0.75	0.87	1.76	-1	124.71
Selous K1	Ilonga	399	2.00	2.27	5.01	-27	298.25
Selous K2	Ilonga	683	1.38	1.80	2.03	-22	111.27
Selous K3	Ilonga	455	0.92	1.19	2.03	-7	147.25
Selous L1	Ilonga	464	1.77	1.59	3.81	-10	221.98
Selous LU1	Ilonga	2443	1.10	1.37	0.45	4	24.56
Selous LU2	Ilonga	1156	1.50	1.31	1.30	-19	70.93
Selous LU3	Ilonga	613	0.60	0.84	0.98	20	45.68
Selous LU4	Ilonga	373	1.43	2.21	3.83	-13	160.86
Selous LU5	Ilonga	510	0.64	0.84	1.26	9	96.08
Selous MB1	Kalulu	2157	2.25	1.48	1.04	4	25.50
Selous N1	Kalulu	1800	1.58	1.00	0.88	-3	26.11
Selous N2	Kalulu	1032	0.69	0.63	0.67	0	28.10
Selous LL2	Kingupira	1272	2.58	1.62	2.03	13	72.33
Selous LL3	Kingupira	1702	2.27	1.19	1.34	-2	47.59
Selous MA1	Kingupira	1684	1.31	0.85	0.78	-11	49.29
Selous MS1	Kingupira	1342	3.18	1.25	2.37	3	58.12
Selous RU1	Kingupira	1786	3.00	1.18	1.68	3	44.79
Selous U3	Kingupira	776	0.71	0.73	0.92	-4	88.92
Selous U4	Kingupira	783	2.82	1.25	3.60	0	94.51
Selous LU6	Likuyu S	883	1.50	1.17	1.70	4	57.76
Selous LU7	Likuyu S	1460	1.17	0.72	0.80	-3	28.08
Selous LU8	Likuyu S	1628	1.17	1.47	0.72	21	23.34
Selous MB2	Likuyu S	1054	2.33	0.98	2.21	1	70.21
Selous MB3	Liwale	1686	0.69	0.75	0.41	6	17.79
Selous MH1	Liwale	1366	0.62	0.65	0.45	0	24.16
Selous ML1	Liwale	792	0.77	0.83	0.97	3	44.19
Selous MT1	Liwale	872	0.75	0.45	0.86	1	55.05
Selous LA1	Matambwe	548	0.64	0.74	1.17	8	94.89
Selous MK1	Matambwe	812	3.23	1.74	3.98	-21	153.94
Selous R3	Matambwe	378	0.57	0.65	1.51	-7	153.44
Selous LL1	Miguruwe	2171	2.46	1.13	1.13	-3	41.46
Selous MJ1	Miguruwe	1935	0.69	0.63	0.36	-1	19.12
Selous MT2	Miguruwe	2018	2.17	1.53	1.07	7	43.61
Selous K4	Msolwa	379	1.50	1.51	3.96	-23	237.47
Selous K5*	Msolwa	592	1.36	1.82	2.29	-42	99.66
Selous M1	Msolwa	432	0.17	0.39	0.39	1	111.11

Selous M2	Msolwa	409	0.79	0.80	1.92	-7	183.37
Selous R1	Msolwa	454	1.69	1.25	3.73	-22	176.21
Selous R2	Msolwa	687	1.15	1.91	1.68	-19	103.35
Selous R4	Msolwa	581	1.67	1.78	2.87	-21	91.22
Selous U1	Msolwa	589	0.92	0.79	1.56	-10	91.68
Selous U2	Msolwa	519	1.58	1.38	3.05	-15	123.31

***** In K5 block, the company stopped lion trophy hunting in 2002 because they were concerned at the high levels of lion hunting in neighbouring blocks (pers. com. Raul Ramoni).

Table B: Data on lion hunting offtake (± standard deviation) and annual rate of change in blocks outside Selous Game Reserve (1996-2008).

Block Name	Area (km^2)	Mean lion shot/block	± SD	Lion Offtake ($lion/1000km^2$)	% Change in Offtake
Gonabisi WMA	702	1.64	1.63	2.33	-14
Kilombero -North combined	2646	1.33	1.09	0.50	-4
Kilombero -South combined+	2882	2.50	2.35	1.73	-99
Kilwa O.A. - Combine	6659	1.39	1.42	0.21	-7
Liwale O.A-North	1292	2.50	1.83	1.94	-2
Liwale O.A-North	2431	2.00	1.28	0.82	-8
Mahenge O.A.	2479	0.93	0.90	0.37	-4
Mbarangandu WMA	4899	1.50	1.20	0.31	-13
Mwambesi G.C.A.	990	1.00	1.10	1.01	9
Mwatisi O.A - Combined	784	1.00	0.00	1.28	-
Ngarambe/Tapika WMA	721	2.00	1.10	2.77	-6
Ruhidji/Ifinga O.A.	1415	1.33	1.37	0.94	-34
Tunduru WMA	2648	1.25	0.96	0.47	-

+ The second largest lion trophy ever recorded was shot in the Kilombero South blocks in the early 2000s and an average of three lions shot annually. However, since then there has been increased conflict with pastoralists, and 22 lions were poisoned in this area in 2005-2006. Since 2006 no lions have been taken from the Kilombero South blocks (pers com. Ryan Shallom).

Table C: Maximum offtake (1996-99), average offtake and quota per block (1996-2008) in SGR

Hunting block	Average quota 1996-2008	Average hunted per block 1996-99	Area (km^2)	Initial Intensity 1996-99 per $1000km^2$	Offtake 1996-2008 per $1000km^2$
Selous IH1	2	0.75	425	1.76	1.76
Selous K1	5	3.50	399	8.77	5.01
Selous K2	4	2.75	683	4.03	2.03
Selous K3	3	1.25	455	2.75	2.03
Selous K4	5	3.25	379	8.58	3.96
Selous K5	4	3.75	592	6.33	2.29
Selous LA1	2	0.50	548	0.91	1.17
Selous L1	5	2.75	464	5.93	3.81
Selous LL1	5	2.00	2171	0.92	1.13
Selous LL2	4	1.75	1272	1.38	2.03
Selous LL3	4	2.50	1702	1.47	1.34
Selous LU1	4	0.75	2443	0.31	0.45
Selous LU2	3	2.50	1156	2.16	1.30
Selous LU3	3	0.25	613	0.41	0.98
Selous LU4	3	3.00	373	8.04	3.83
Selous LU5	3	0.25	510	0.49	1.26
Selous LU6	4	1.00	883	1.13	1.70
Selous LU7	3	1.50	1460	1.03	0.80
Selous LU8	3	0.25	1628	0.15	0.72
Selous M1	3	0.25	432	0.58	0.39
Selous M2	4	1.00	409	2.44	1.92
Selous MA1	4	2.00	1684	1.19	0.78
Selous MB1	4	1.75	2157	0.81	1.04
Selous MB2	4	2.00	1054	1.90	2.21
Selous MB3	2	0.50	1686	0.30	0.41
Selous MH1	1	0.75	1366	0.55	0.45
Selous MJ1	1	0.75	1935	0.39	0.36
Selous MK1	5	4.00	812	4.93	3.98
Selous ML1	2	0.50	792	0.63	0.97
Selous MS1	5	3.75	1342	2.79	2.37
Selous MT1	1	0.50	872	0.57	0.86
Selous MT2	4	1.00	2018	0.50	1.07
Selous N1	4	1.75	1800	0.97	0.88
Selous N2	2	0.50	1032	0.48	0.67
Selous R1	4	2.75	454	6.06	3.73
Selous R2	4	2.50	687	3.64	1.68
Selous R3	2	0.75	378	1.98	1.51
Selous R4	4	2.50	581	4.30	2.87

Selous RU1	5	3.25	1786	1.82	1.68
Selous U1	3	1.75	589	2.97	1.56
Selous U2	3	2.50	519	4.82	1.38
Selous U3	5	1.00	776	1.29	0.92
Selous U4	4	3.00	783	3.83	3.60

Raw data on lion hunting offtake and quotas for Tanzania's hunting blocks is available from the CITES office at the Wildlife Division headquarters in Dar es Salaam, Tanzania. Lion trophy export data is available from the CITES trade database website. See: http://trade.cites.org/

Figure A. Average number of lions shot per block in Selous Game Reserve and the average lion hunting quota per block per year.

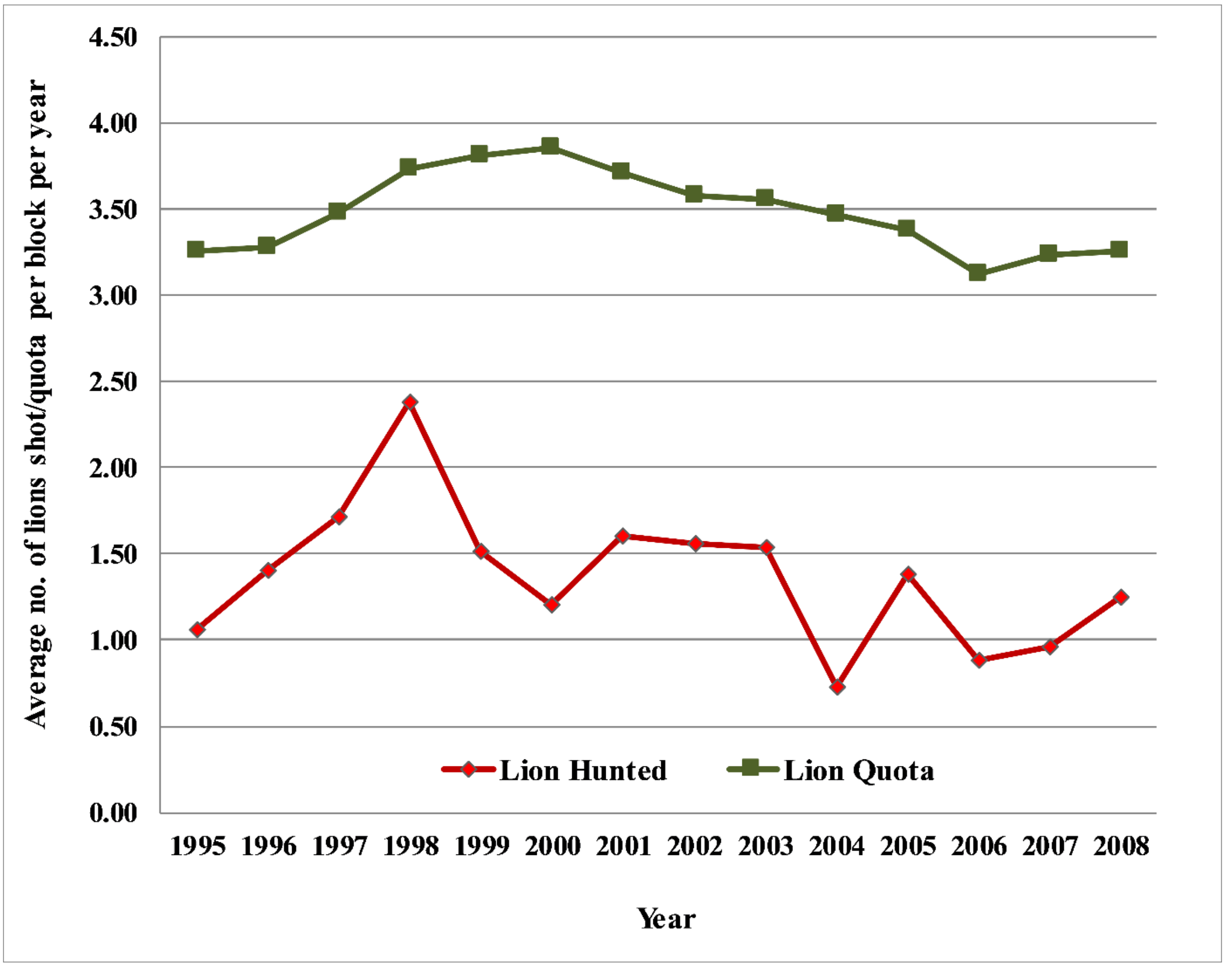

Note that quotas are far higher than offtake.
